# 3D-Printed Gelatin Methacrylate Scaffolds with Controlled Architecture and Stiffness Modulate the Fibroblast Phenotype towards Dermal Regeneration

**DOI:** 10.3390/polym13152510

**Published:** 2021-07-30

**Authors:** Rita I. R. Ibañez, Ronaldo J. F. C. do Amaral, Rui L. Reis, Alexandra P. Marques, Ciara M. Murphy, Fergal J. O’Brien

**Affiliations:** 1 Tissue Engineering Research Group, Department of Anatomy and Regenerative Medicine, Royal College of Surgeons in Ireland, D02 YN77 Dublin, Ireland; ritaibanez@rcsi.ie (R.I.R.I.); ronaldoamaral@rcsi.ie (R.J.F.C.d.A.); 2 3B’s Research Group, I3Bs—Research Institute on Biomaterials, Biodegradables and Biomimetics, Headquarters of the European Institute of Excellence on Tissue Engineering and Regenerative Medicine, AvePark–Parque de Ciência e Tecnologia, University of Minho, 4710-057 Braga, Portugal; rgreis@i3bs.uminho.pt (R.L.R.); apmarques@i3bs.uminho.pt (A.P.M.); 3 ICVS/3B’s—PT Government Associate Laboratory, 4710-057 Braga, Portugal; 4 Trinity Centre for Biomedical Engineering, Trinity Biomedical Sciences Institute, Trinity College Dublin, D02 YN77 Dublin, Ireland; 5 Advanced Materials and Bioengineering Research (AMBER) Centre, Royal College of Surgeons in Ireland, D02 YN77 Dublin, Ireland

**Keywords:** biomaterial stiffness, porosity, wound healing, GelMA, 3D printing, fibroblast, fibrosis inhibition

## Abstract

Impaired skin wound healing due to severe injury often leads to dysfunctional scar tissue formation as a result of excessive and persistent myofibroblast activation, characterised by the increased expression of α-smooth muscle actin (αSMA) and extracellular matrix (ECM) proteins. Yet, despite extensive research on impaired wound healing and the advancement in tissue-engineered skin substitutes, scar formation remains a significant clinical challenge. This study aimed to first investigate the effect of methacrylate gelatin (GelMA) biomaterial stiffness on human dermal fibroblast behaviour in order to then design a range of 3D-printed GelMA scaffolds with tuneable structural and mechanical properties and understand whether the introduction of pores and porosity would support fibroblast activity, while inhibiting myofibroblast-related gene and protein expression. Results demonstrated that increasing GelMA stiffness promotes myofibroblast activation through increased fibrosis-related gene and protein expression. However, the introduction of a porous architecture by 3D printing facilitated healthy fibroblast activity, while inhibiting myofibroblast activation. A significant reduction was observed in the gene and protein production of αSMA and the expression of ECM-related proteins, including fibronectin I and collagen III, across the range of porous 3D-printed GelMA scaffolds. These results show that the 3D-printed GelMA scaffolds have the potential to improve dermal skin healing, whilst inhibiting fibrosis and scar formation, therefore potentially offering a new treatment for skin repair.

## 1. Introduction

The skin is the largest organ of the human body, which forms an effective barrier against the external environment and protects the body from dehydration and environmental insults [1,2]. Upon injury, the skin wound-healing process begins immediately via a dynamic series of physiological events to repair and restore structural integrity and function to the damaged site. However, in extreme cases, such as severe burns, this repair process is disrupted and can result in fibrotic scar formation, characterised by the abnormal deposition of highly dysfunctional tissue [3]. In such cases, skin autografts are required, but this treatment option is often limited due to unavailability of healthy donor tissue. Furthermore, the use of skin grafts often results in excessive scarring and the challenges associated with scarring, such as loss of tissue function, contracture, limited mobility and pain [1]. 

The extracellular matrix (ECM) is actively involved in both cellular and extracellular events that lead to scar formation. The dermis layer of the skin is composed of the ECM, separated from the epidermis by the basement membrane [4]. Dermal fibroblasts play a key role in the wound-healing process by synthesising and remodelling the ECM through the production of a number of proteins, including collagens and fibronectin. During this process, fibroblasts transition to myofibroblasts, which contract and close the wound. However, excessive and persistent myofibroblast activation can impede regeneration to a level comparable to the original tissue [5,6]. Fibroblast-to-myofibroblast differentiation is mediated by a variety of inflammatory factors and mechanical stimuli [7,8]. Myofibroblasts are characterised by their elongated morphology; expression of α-smooth muscle actin (αSMA); enhanced contractility and increased production of ECM proteins, such as fibronectin I (FN); and collagen I (Col I) and collagen III (Col III) when compared to normal fibroblasts [9,10]. Extensive research has led to an in-depth knowledge of the mechanism of impaired wound healing, which leads to fibrosis and scarring [11], and the field of tissue-engineered skin substitutes has progressed over a number of years [12]. Yet, despite these advances, preventing scar formation and contracture in skin wound healing remains a significant challenge [13,14]. As such, there is a real clinical need for alternative approaches to skin wound healing that induce regeneration upon injury, while preventing scar formation. Targeting the activity of dermal fibroblasts during wound repair through optimised scaffold design may provide a new means of achieving this.

Biomimetic-tissue-engineering-based approaches have shown some potential in this field, yet a number of technological limitations have impeded their success. A significant challenge is the development of biomaterials that can withstand the elasticity, flexibility and stretching that native skin is subjected to while supporting the multiple distinct layers found in the tissue [15]. Furthermore, controlling pore size and interconnectivity, thickness and layer positioning is often difficult to achieve [16]. As such, wound contraction remains a major issue in scaffold-driven dermal regeneration, and the prevention of scar formation has yet to be achieved. 3D printing has recently emerged as a potential method of addressing these challenges as it allows for the development of high-definition scaffolds with controlled mechanical and structural properties, for improved cell and tissue integration, to enhance skin regeneration [17,18]. Furthermore, the rate of production possible with 3D printing allows for more rapid and efficient treatments [19]. More recently, 3D freeform printing systems have successfully omnidirectionally 3D-printed nanocomposite hydrogel scaffolds. This technique has improved conventional layer-by-layer 3D printing by allowing the production of complex biomimetic designs [20].

Collagen is the main structural protein in skin, and its biocompatibility and low immunogenicity make it an ideal biomaterial for skin tissue applications. However, its use in 3D printing is challenging due to the processing required for printing and its low handling and mechanical properties [21]. A common alternative to collagen is the use of gelatin, a derivative from the degradation of collagen, which has desirable gelling properties, biocompatibility and biodegradability [22]. Although gelatin-based hydrogels are easily 3D-printed, their usage in vitro is limited by poor thermal stability. This limitation can be overcome by applying chemical or physical cross-linking. One such example is methacrylate gelatin (GelMA), an engineered gelatin-based material that has been proven to be versatile for a number of biomedical applications due to its suitable biological properties and tuneable physical characteristics [23,24]. Its application in 3D printing makes it possible to create on-demand high-resolution scaffolds with controlled pore size and architecture as well as defined interconnectivity between the pores, suitable for skin tissue engineering [25]. 

With this in mind, this study investigated the effect of GelMA stiffness and porosity on fibroblast behaviour, with the specific aim of designing and characterising a range of 3D-printed GelMA scaffolds with different pore sizes and porosity to promote dermal fibroblast activity, while reducing myofibroblast activation. 

## 2. Materials and Methods

### 2.1. Materials

Unless stated otherwise, all reagents were purchased from Sigma-Aldrich, Ireland. AlamarBlue and Pico Green were purchased from Biosciences, Cambridge, UK. The live/dead assay kit was purchased from Invitrogen, Oxford, UK. Monoclonal antibodies were purchased from Abcam, Cambridge, UK. Methacrylate gelatin (GelMA) was purchased from CellSystems, Troisdorf, Germany. 30G needles were purchased from ATS Adhesives Limited, Dublin, Ireland, and 24-well-plate cell inserts were purchased from VWR, Dublin, Ireland. The RNeasy extraction kit and Qiagen Quantitec primers were purchased form Qiagen, Manchester, UK. Adult human dermal fibroblasts (HdFs) (Detroit 551—ATCC^®^ CCL-110™) were purchased from ATCC, Manassas, Virginia, USA. Transforming growth factor β1 (TGF-β1) was purchased from PeproTβech, London, UK.

### 2.2. The Effect of GelMA Mechanical Stiffness on Myofibroblast Activation and Differentiation

#### 2.2.1. GelMA Hydrogel Fabrication and Mechanical Characterisation

A range of GelMA hydrogels of 1%, 1.5%, 2%, 3%, 8% and 10% (*w/v*) were prepared by dissolving the corresponding amount of GelMA into 0.5% (*w/v*) lithium phenyl-2,4,6-trimethylbenzoylphosphinate (LAP) in PBS solution at 60 °C and maintained at 37 °C until fully dissolved [26]. The final solution was poured into 24-well-plate cell inserts. The well plates were kept at 4 °C for 30 min and then UV (405 nm) was applied for 3 min for cross-linking. The range of GelMA hydrogels was mechanically tested in unconfined compression using a standard testing machine with a 5N load cell (Zwick Roell Z005, Zwick Roell, Ulm, Germany). Briefly, the hydrogels were kept hydrated through immersion in a PBS bath maintained at room temperature. A preload of 0.01 N was applied to ensure that the hydrogel surface was in direct contact with the impermeable loading platens, with 10% strain. Hydrogels with mechanical stiffness in the range of soft (<5 kPa), medium (~10 kPa) and stiff (>20 kPa) were chosen for further cell studies, as stiff environments are known to induce myofibroblast activation. 

#### 2.2.2. Assessing Fibroblast Morphology and Phenotype on GelMA Hydrogels

Human dermal fibroblasts (HdFs) were cultured in T175 culture flasks using low-glucose DMEM optimised with 10% FBS and 5% P/S. Before seeding, cells were detached using trypsin and suspended at a concentration of 10^7^ cells/mL. The hydrogels were seeded with 2 × 10^4^ and 1 × 10^4^ HdFs per hydrogel for gene expression and immunohistochemistry assays, respectively. The 24-well plates were then placed in an incubator for 3 h to allow initial cellular attachment. After the incubation period, 1 mL of 2% FBS and 1% P/S low-glucose DMEM were added to each well and the plates were returned to the incubator. As a positive control, 15 ng/mL of transforming growth factor β1 (TGF-β1), which has been shown to induce myofibroblast activation [27], 2% FBS and 1% P/S low-glucose DMEM were used. Both culture flasks and seeded scaffolds were cultured under standard conditions (37 °C, 5% CO_2_). The media were changed every 2 days. 

After 7 days, samples were collected for gene expression analysis. Total RNA was isolated using an RNeasy extraction kit according to the manufacturer’s instructions. Samples cultured with soft gel and without TGF-β1 were used as controls. Target mRNAs analysed were COL1A1, COL3A1, FN1, αSMA and CTGF, with GAPDH used as a housekeeping gene. The list of primers that were used for the amplification of these genes is listed in Table 1.

To assess αSMA protein expression, scaffolds were fixed in 10% formalin for 15 min at room temperature and then transferred into PBS at 4 °C. To identify the HdF actin cytoskeleton on the hydrogel scaffolds, the antigen was identified with Tritc-phalloidin (1:500). The samples were also stained for αSMA with monoclonal (1:100) and goat anti-rabbit IgG H&L Alexa Fluor 564 (1:250) antibodies. Cell nuclei were stained with DAPI (dilution 1:1000). Imaging and quantification were carried out using Zeiss 710 NLO, (ZEISS, Jena, Germany) and Image J, respectively. To evaluate cell morphology, the cell spread area and circularity were measured using Image J measuring tools. The circularity was calculated following Equation (1), in which the value 1.0 indicates a perfect circle.

**Equation (1):** Circularity

(1) $$4\times \pi \;\left(\frac{Area}{Perimeter^{2}}\right)$$

### 2.3. Development of 3D-Printed GelMA Scaffolds for Dermal Regeneration

#### 2.3.1. Fabrication of 3D-Printed Scaffolds 

To create porous GelMA scaffolds, an Allevi II (Allevi, Philadelphia, PA, USA) 3D printer was used to print GelMA, coupled with a 10 mL syringe and a 30 G needle. Then, 10% (*w/v*) GelMA was dissolved in 0.5% (*w/v*) lithium phenyl-2,4,6-trimethylbenzoylphosphinate (LAP) in PBS at 60 °C and maintained at 37 °C until fully dissolved [26]. The solution was then poured into a 10 mL syringe and stored at 4 °C until ready for use. To 3D-print GelMA, a code containing all the toolpaths for the prints (G-code), including printing speed and GelMA deposition, inter-filament distance, structure perimeter, cross-linking time and cross-linking intensity to have exact control of the 3D printing process, was developed using Python, a high-level and general-purpose programming language. Four designs were then developed, based on inter-filament spacings, in order to create different pore sizes. From designs 1 to 4, the inter-filament spacings were 2, 1, 0.75 and 0.6 mm, respectively. The 3D printing parameters are presented in Table 2. Pressure between 10 and 20 PSI was applied at a temperature of 25–26 °C. Photo-cross-linking was carried out by exposing the GelMA bioink to blue light (405 nm) for 3 min after printing. All designs were kept within a closed 24-well plate at 4 °C until further use.

#### 2.3.2. Characterisation of 3D-Printed GelMA Scaffolds 

A number of methods were used to characterise the 3D-printed designs developed. Pore and filament analyses were conducted using a microscope (Nikon 90i) and Image J software. The degree of swelling (DS) was calculated according to Equation (2) using the weights of the samples in dry and wet conditions for 0.5, 1, 3, 5 and 24 h in PBS at 37 °C.

**Equation (2):** Degree of swelling equation (DS). Ws corresponds to the swollen weight and Wd to the dry weight.

(2) $$DS\;\left(\%\right)=\frac{Ws-Wd}{Ws}\times 100$$

The overall porosity percentage of the samples was also calculated according to the following equation (Equation (3)). Scaffold density was calculated based on solid density.

**Equation (3):** Percentage porosity

(3) $$Porosity\;\left(\%\right)=1-\frac{Scaffold\;density}{Solid\;density}\times 100$$

Scaffolds were mechanically tested in unconfined compression using a standard testing machine with a 5N load cell (Zwick Roell Z005). Briefly, scaffolds were kept hydrated through immersion in a PBS bath maintained at room temperature. A preload of 0.01 N was applied to ensure that the scaffold surface was in direct contact with the impermeable loading platens. Five cycles of unconfined uniaxial compression relaxation tests were performed with 10% strain.

#### 2.3.3. Fibroblast Behaviour on 3D-Printed GelMA Scaffolds

The 3D-printed scaffolds were sterilised with 70% ethanol and UV exposure, followed by PBS washes to remove traces of ethanol. The scaffolds were placed in 24-well plates, one scaffold per well, and seeded with 33.4 µL of the cell suspension with 5 × 10^5^ HdFs. The 6-well plates were then placed in an incubator for 3 h to allow initial cell attachment. After the incubation period, 1 mL of supplemented medium (low-glucose DMEM with 10% FBS) was added to each well and the plates were returned to the incubator. Both culture flasks and seeded scaffolds were cultured under standard conditions (37 °C, 5% CO_2_).

Cell metabolic activity was assessed at 1, 3 and 7 days post-seeding using AlamarBlue solution according to the manufacturer’s instructions. In short, 10% AlamarBlue solution was made in standard culture media. Fluorescence was read at 560/590 nm (excitation/emission). 

To assess cell proliferation, DNA content within the scaffolds was evaluated at 1, 3 and 7 days post-seeding using a Quant-iTTM Pico-Green dsDNA kit. Scaffolds were placed in Eppendorf containing 1 mL of lysis buffer. The samples then underwent three freeze/thaw cycles at −80 °C before the assay was performed per the manufacturer’s instructions. The DNA concentration was determined using a standard curve. A live/dead assay for cell viability was also carried out 3 and 7 days after incubation using a live/dead kit and the staining observed under the microscope (Eclipse Nikon 90i, Nikon, Tokyo, Japan). Live cells were stained green, and dead cells were stained red.

Cell culture was carried out like hydrogel cell culture but with the range of scaffolds created by 3D-printing GelMA, and 7 days post-seeding, samples were collected for immunocytochemical and gene expression analysis. Immunocytochemical and gene expression analysis was performed as previously described.

### 2.4. Statistical Analysis

Statistical analyses were performed using GraphPad Prism (version 5) software with 3–4 samples analysed for each experimental group. Pairwise comparisons between means of different groups were performed using Student’s *t*-test. Two-way ANOVA was used for analysis of variance, with Tukey’s post hoc test to compare between groups. Numerical and graphical results were displayed as the mean ± standard deviation. Significance was accepted at a level of *p* < 0.05.

## 3. Results and Discussion

Impaired skin wound healing due to severe injury often leads to the formation of fibrotic and dysfunctional scar tissue. Myofibroblasts play a key role in wound healing. However, excessive and persistent action can result in undesirable contracture and scarring. Myofibroblast activation is regulated by several key cytokines, such as transforming growth factor β1 (TGF-β1), which promote persistent activation via a positive regulation loop [28,29]. This process is often characterised by the increased expression of αSMA and ECM proteins [30,31]. Yet, despite extensive research on impaired wound healing, scar formation and contracture remain a challenge [32]. Therefore, the aim of this study was to investigate the mechanical effect of GelMA stiffness on human dermal fibroblast behaviour and develop 3D-printed GelMA scaffolds with tuneable pore size and porosity that would support fibroblast activity but inhibit myofibroblast activation, reducing the potential for scar tissue formation.

### 3.1. GelMA Hydrogel Fabrication and Mechanical Characterisation

**Figure 1:**
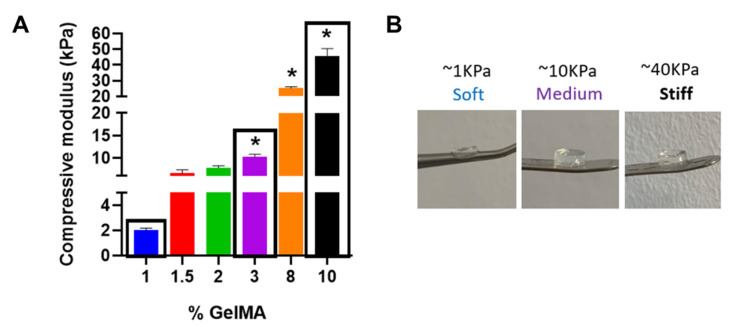
(**A**) Effect of increasing concentrations of GelMA on the compressive modulus of the hydrogels. The compressive modulus increased with increasing GelMA concentration. Hydrogels containing 3%, 8% and 10% GelMA had a significantly higher compressive modulus compared with 1% GelMA hydrogels (* *p* < 0.05). (**B**) Images of selected soft (2%), medium (3%) and stiff (10%) GelMA hydrogels.

We first assessed the effect of the mechanical properties of bulk GelMA hydrogels on fibroblast activity to understand the role of GelMA stiffness in myofibroblast activation. Fibroblasts tune their morphology and cytoskeletal structure in response to matrix stiffness [33], and studies have shown that mechanical stimuli can trigger myofibroblast activation, with matrix stiffness greater than 20 KPa effective in driving the phenotype transition of fibroblasts to myofibroblasts [34,35]. This activation is linked with faster wound closure [36], which is important for initial wound healing. However, long-term activation can lead to fibrosis in later stages of the wound-healing response, impairing skin regeneration by inducing scar formation [37]. This activation has been attributed to the stiffness of the substrate, whereby increased stress signals increase myofibroblast activation and survival [38]. To determine whether the same effect of GelMA stiffness can be attributed to myofibroblast transition, bulk GelMA hydrogels were initially fabricated with mechanical properties that ranged from soft (<3 kPa) to stiff (>20 kPa), using different concentrations of GelMA with a constant concentration of a photoinitiator (LAP) of 0.5% (*w/v*) (Figure 1A). 

As expected, increasing the concentration of GelMA significantly increased the mechanical properties of the hydrogels from ~3 up to ~40 kPa. Hydrogels containing 3%, 8% and 10% GelMA had a significantly higher compressive modulus than 1% GelMA hydrogels (*p* < 0.05). As such, hydrogels with GelMA concentrations of 1%, 3% and 10% were taken forwards as representative of significantly different soft (~3 kPa), medium (~10 kPa) and stiff (~40 kPa) hydrogels for further analysis with human dermal fibroblasts to elucidate the effect of stiffness on human dermal fibroblast behaviour. 

### 3.2. Effect of GelMA Hydrogel Stiffness on Fibroblast Morphology and Phenotype

**Figure 2:**
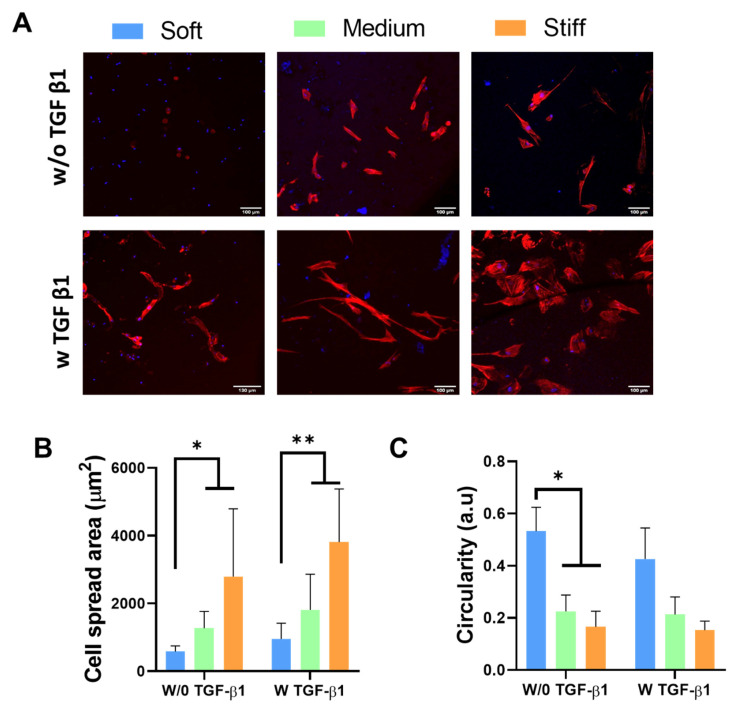
Effect of increasing GelMA hydrogel stiffness on (**A**) cell morphology, (**B**) cell spread area and (**C**) cell circularity. * *p* < 0.05 compared to the soft GelMA hydrogel in the absence of TGF-β1; ** *p* < 0.05 compared to the soft GelMA hydrogel in the presence of TGF-β1.

Changes in cell morphology have previously been reported to control a variety of cell behaviours, including cell division [39], proliferation and apoptosis [40], migration [41] and differentiation [42]. Studies have reported that during fibroblast-to-myofibroblast transition, cells undergo morphological changes, most notably becoming elongated in shape, with a larger cell spread area and reduced circularity [5,43]. We investigated fibroblast cell morphology on the soft (~3 kPa), medium (~10 kPa) and stiff (~40 kPa) GelMA hydrogels to determine whether the range of GelMA stiffness achieved would induce morphological changes in the seeded dermal fibroblasts (Figure 2). In addition, myofibroblast activation is regulated by several key cytokines, such as transforming growth factor-β1 (TGF-β1), which promote persistent activation via a positive regulation loop [28,29]. Thus, TGF-β1 was used as a positive control for myofibroblast activation. Figure 2A demonstrates significant morphological changes in the fibroblasts across the GelMA hydrogel stiffness range, with cells taking on a more elongated morphology in the stiffer hydrogels. In addition, the cell spread area significantly increased and cell circularity significantly decreased with increasing stiffness (*p* > 0.05) (Figure 2B,C). Furthermore, the addition of TGF-β1 did not affect the spread area or circularity of the cells on the hydrogels, indicating that the stiffness of GelMA alone is sufficient to induce morphological changes indicative of myofibroblast differentiation. Nevertheless, this is expected because although the stiffness of the materials promotes changes, it is not expected to “exhaust” the cells. Therefore, they are still capable of responding to TGF-β1.

A hallmark of the myofibroblast phenotype is the expression of αSMA, a cytoskeletal protein that promotes increased force production, contributing to wound closure. However, excessive expression of αSMA can lead to undesirable wound contracture and scarring [29,44].

**Figure 3:**
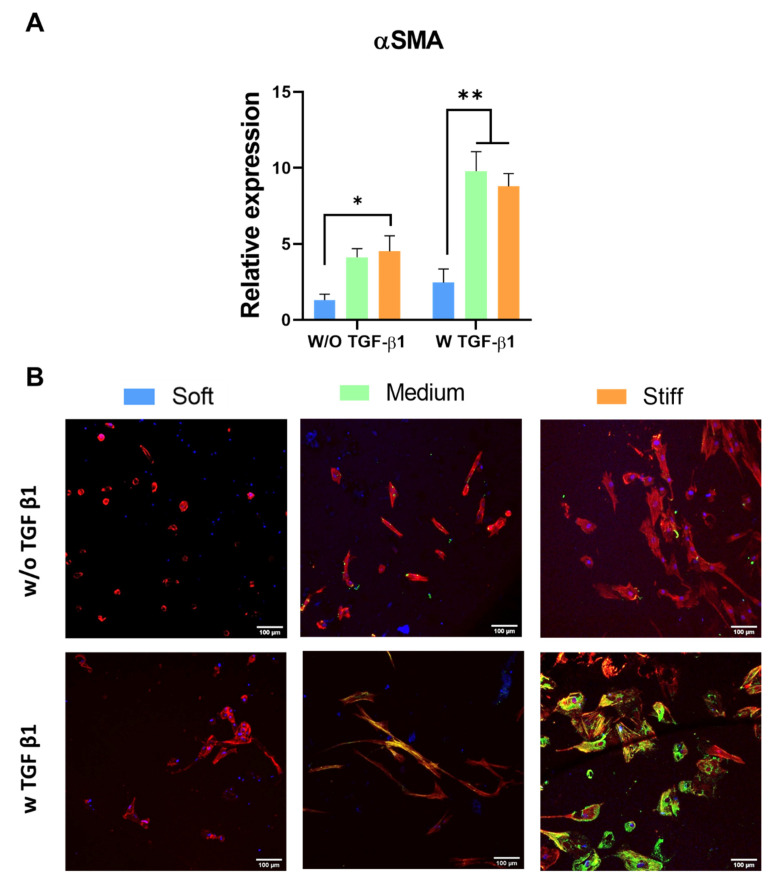
Effect of increasing GelMA hydrogel stiffness on αSMA gene expression (* *p* < 0.05 compared to the soft GelMA hydrogel in the absence of TGF-β1; ** *p* < 0.05 compared to medium and stiff GelMA hydrogels in the presence of TGF-β1) (**A**) and αSMA protein expression (**B**). The addition of TGF-β1 enhances the production of α-SMA protein. DAPI (blue), phalloidin (red) and αSMA (green). Scale bar = 100 µm.

Figure 3A shows that increasing GelMA stiffness promoted increased gene expression of αSMA, even in the absence of TGF-β1, with the highest level of expression observed in the stiff GelMA hydrogel (*p* < 0.05). Whilst the addition of TGF-β1 did not affect the expression of αSMA in the soft GelMA hydrogels, the increases observed in the medium and stiff hydrogels were significantly enhanced in the presence of TGF-β1 (*p* < 0.05). These results are confirmed in Figure 3B, which shows increasing αSMA protein expression with increasing GelMA hydrogel stiffness in the presence of TGF-β1, with the stiff GelMA hydrogel promoting the greatest level of αSMA protein production. 

Taken together, these results demonstrate that increasing stiffness of GelMA hydrogels promotes myofibroblast activation and fibrosis-related gene and protein expression. This effect was greater in the presence of TGF-β1, indicating a synergistic effect of TGF-β1 and GelMA stiffness in stimulating myofibroblast differentiation, as reported previously [45].

**Figure 4:**
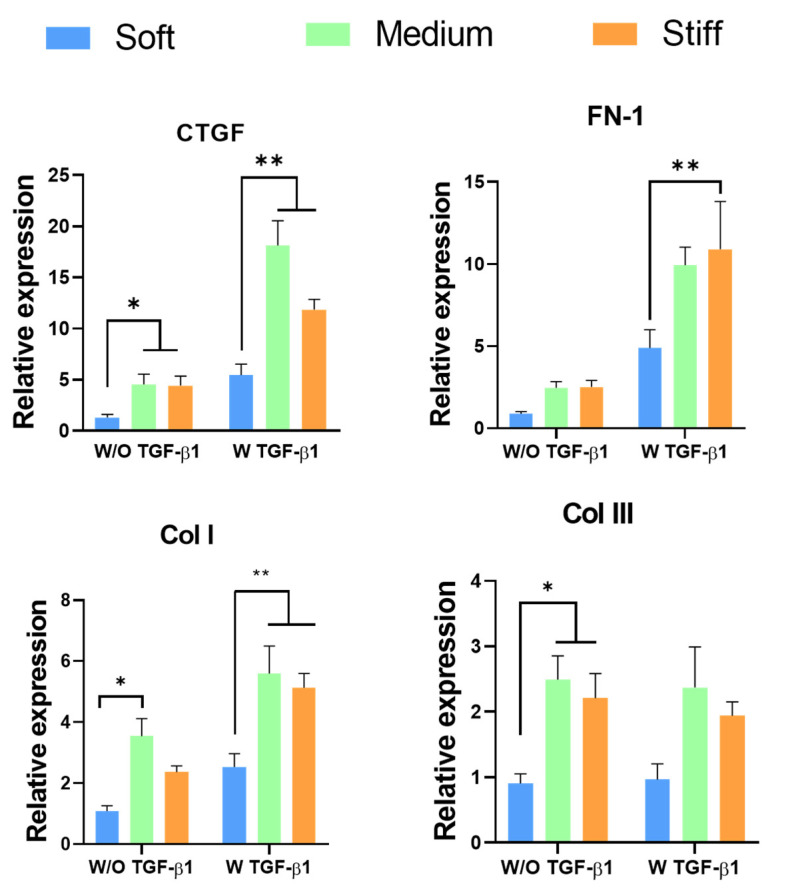
Effect of increasing GelMA hydrogel stiffness on connective tissue growth factor (CTGF), fibronectin I (FN), collagen I (Col I) and collagen III (Col III) expression. * *p* < 0.05 compared to the soft GelMA hydrogel in the absence of TGF-β1; ** *p* < 0.05 compared to the soft GelMA hydrogel in the presence of TGF-β1.

Connective tissue growth factor (CTGF) is a central mediator of tissue remodelling and fibrosis. It activates myofibroblasts and stimulates the deposition and remodelling of ECM proteins, including fibronectin I (FN), collagen I (Col I) and collagen III (Col III) [21,27,31]. A number of studies have shown that increasing mechanical properties of the ECM influences the expression of these proteins and promotes myofibroblast activation [46,47]. We therefore sought to determine whether GelMA stiffness would influence myofibroblast activation in a similar manner. CTGF gene expression was significantly up-regulated in both medium and stiff GelMA hydrogels compared with the soft GelMA hydrogel (Figure 4). The increase was more pronounced in the TGF-β1-treated groups. The expression of FN increased in the medium and stiff hydrogels in the absence of TGF-β1, although the difference in the expression levels was not significant. However, in the presence of TGF-β1, the expression of FN significantly increased with increasing stiffness. Expression of Col I was significantly up-regulated in the untreated GelMA hydrogel. Interestingly, this significant increase was not observed in the stiff hydrogels. However, in the presence of TGF-β1, a significant increase in Col I expression was observed in the medium and stiff GelMA hydrogels compared with the soft GelMA hydrogel. Col III gene expression was significantly up-regulated in the medium and stiff GelMA hydrogels without the addition of TGF-β1. 

### 3.3. Fabrication and Characterisation of 3D-Printed GelMA Scaffolds 

In the literature, there is extensive research highlighting a porous architecture as an important consideration in the design of biomaterials for tissue regeneration [30,48]. 3D printing technology allows rapid and controlled fabrication of biomaterials with a precise and defined porous architecture. Having demonstrated that GelMA stiffness affects fibroblast morphology and gene expression of ECM and myofibroblast activation proteins, we investigated whether 3D-printed GelMA scaffolds with a defined porous architecture could reduce the effect of bulk-GelMA-hydrogel-stiffness-induced myofibroblast activation and better control dermal fibroblast behaviour towards scarless skin regeneration. Although bulk GelMA hydrogel stiffness results showed the softer hydrogels (~3 kPa) to be optimal for inhibiting myofibroblast activation, it was not possible to use this concentration for 3D printing. Recently, Shie et al. (2020) investigated a range of 3D-printed GelMA scaffolds (5–15% GelMA concentration) to determine the optimal GelMA concentration for 3D printing and reported that concentrations of 10% and 15% GelMA are optimal for fabrication as concentrations below this have a sol–gel temperature below room temperature [48]. Therefore, we fabricated 3D-printed scaffolds using 10% GelMA concentration, the same concentration as the stiffest GelMA hydrogel, and developed four different scaffold designs by varying the intra-filament distance. A number of design parameters were evaluated, including resulting pore size, porosity, mechanical properties and swelling. 

**Figure 5:**
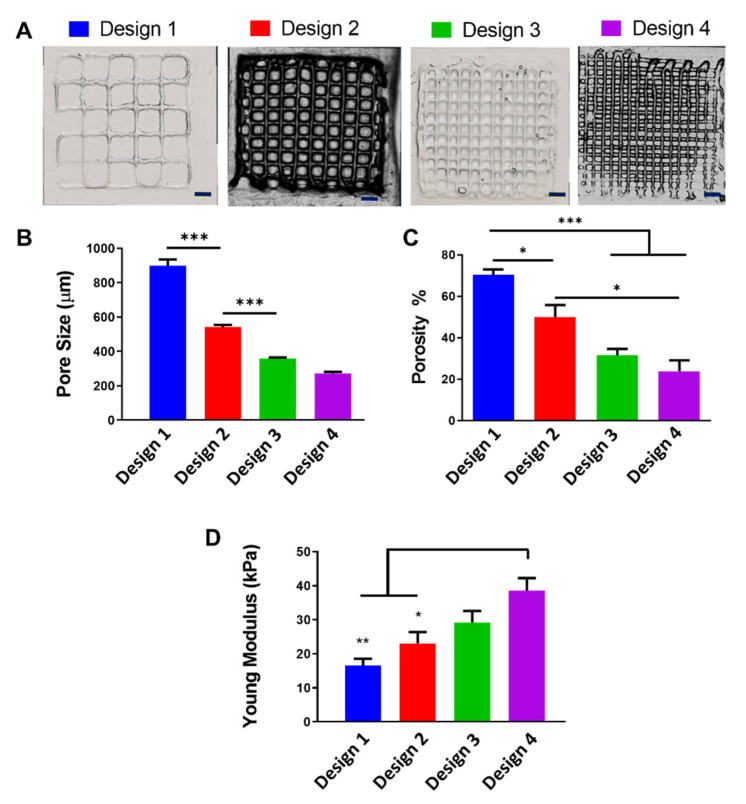
Altering intra-filament distance produced four scaffold designs (**A**). The effect of intra-filament distance on the pore size (**B**), porosity (**C**) and compressive modulus (**D**) of the scaffolds. * *p* < 0.05; ** *p* < 0.001; *** *p* < 0.0001 compared to design 1. Scale bar = 1000 µm.

Figure 5A shows representative images of the four 3D-printed designs, referred to as designs 1–4, with intra-filament distances of 2, 1, 0.75 and 0.6 mm, respectively. Results demonstrated that pore size is dependent on intra-filament distance and decreases with reducing distance. Design 1 had the largest pore size of 898.6 µm, and designs 2, 3 and 4 had pore sizes of 541.7, 358.1 and 272.03 µm, respectively (Figure 5B). Pore size has a significant effect on cell behaviour, and the optimal pore size range is dependent on the cell type, biomaterial and tissue-specific applications [49,50]. In the field of skin healing, a number of studies have investigated the effect of pore size on fibroblast behaviour. One such study investigated human dermal fibroblast activity on 3D-printed gelatin scaffolds with pores ranging from 400 to 750 µm and found that scaffolds with pores larger than 580 µm support increased cell viability and proliferation compared with scaffolds with smaller pore sizes [51]. Another study, by Parenteau-Bareil et al., demonstrated that freeze-dried collagen scaffolds with pore sizes ranging from 130 to 200 µm support human dermal fibroblast adhesion and proliferation over a long-term culture period of 35 days [52]. The pore sizes produced in all four scaffold designs are considered within the optimal range for fibroblast viability, proliferation and migration.

Similar to pore size, reduction in the intra-filament distance decreased the porosity. Scaffold porosity was calculated based on the material’s density (10 g/100 mL). Figure 5C shows the different porosities achieved by altering the intra-filament distance. Design 1 was the most porous scaffold, at 70.4%; designs 2, 3 and 4 had a porosity of 49.93%, 31.53% and 23.86%, respectively. It is generally accepted that the higher the porosity, the better the construct development. However, there is an important balance, as an increase in porosity will lead to a decrease in mechanical properties. This is generally a greater concern in natural biomaterials as they are mechanically weaker materials. With this in mind, the effect of macroporosity on the mechanical properties of the scaffolds was investigated using an unconfined uniaxial compression relaxation test. Results showed that designs 1, 2, 3 and 4 have a compressive modulus of 16.51, 23.08, 29.27 and 38.63 kPa, respectively (Figure 5D), demonstrating that reducing the porosity increases the mechanical properties. Although a concentration of 10% GelMA was used for all four scaffold designs, the mechanical properties of the 3D-printed GelMA scaffolds were lower than the compressive modulus of the bulk 10% GelMA hydrogel. 

**Figure 6:**
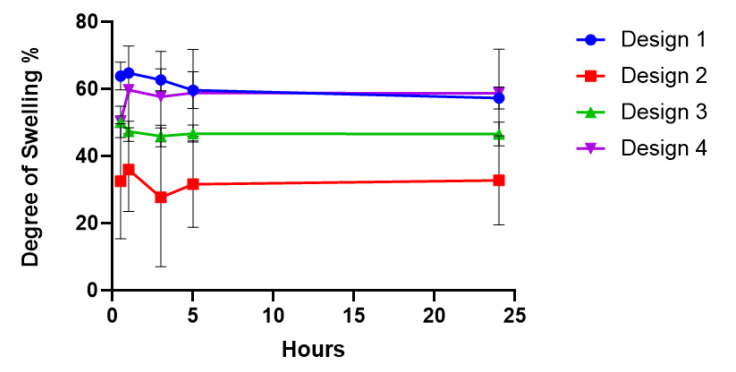
Degree of GelMA hydrogel swelling over 24 h in each of the four scaffold designs.

Hydrogel and biomaterial swelling is an essential consideration since it affects various parameters, including surface properties, cell mobility and waste diffusion, as the hydrogel swelling increases the area permitted for diffusion across the hydrogel network [53,54]. Swelling is also beneficial in the wound-healing process as it builds a small amount of pressure on the wound boundaries [55]. This may increase surface contact with this tissue, contributing to decreased wound contraction and, as a result, stimulating cell ingrowth and therefore wound healing [55,56]. Due to the changes in the porous architecture across the design range, the swelling ability of the 3D-printed GelMA scaffolds was investigated and found to be independent of pore size or porosity (Figure 6). All designs rapidly absorbed water, with over 30% uptake observed in all designs after only 30 min. No significant difference in swelling behaviour was observed between the four scaffold designs, for up to 24 h, indicating that porosity did not affect the swelling capacity of the scaffolds. The concentrations of GelMA and LAP were consistent in all scaffold designs; therefore, the number of cross-links within the matrix remained unchanged, maintaining equal fluid kinetics in all four scaffold designs [57]. 

Our results demonstrated that by altering intra-filament distance, we successfully maintained high resolution and definition during fabrication, producing 3D-printed GelMA scaffolds with a controlled porous architecture and mechanical properties without altering scaffold composition or swelling properties.

### 3.4. Effect of 3D-Printed GelMA Scaffolds on Fibroblast Behaviour

**Figure 7:**
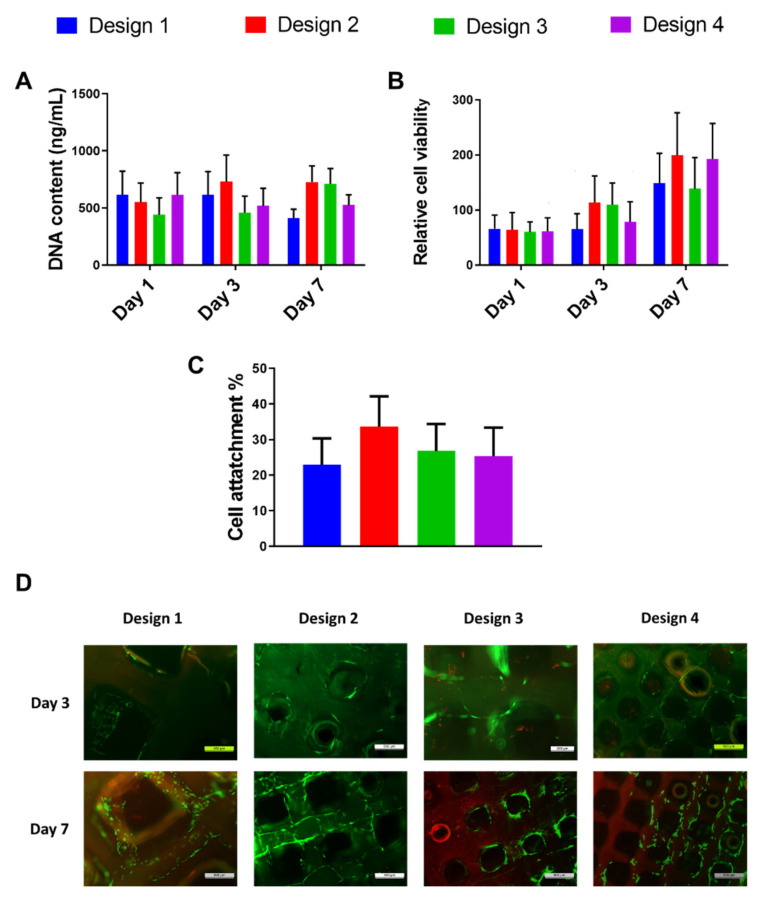
Effect of GelMA scaffold porous architecture on human dermal fibroblast DNA content (**A**), relative cell viability (**B**), attachment (**C**) and live/dead cell assay (**D**). Live cells are stained in green, and dead cells are stained red. No statistical difference was found between groups.

As previously mentioned, a scaffold porous architecture and mechanical properties can significantly affect cell behaviour. We investigated fibroblast growth and morphology on the developed range of 3D-printed GelMA scaffolds to determine the effect of a porous architecture on fibroblast behaviour. All four scaffold designs supported fibroblast attachment, viability and proliferation over 7 days of culture (Figure 7). However, there was no significant difference observed in cell attachment (Figure 7A), viability (Figure 7B) or DNA content (Figure 7C) between the different designs, indicating that normal dermal fibroblast activity within the 3D-printed GelMA scaffolds is independent of pore size and porosity. Figure 7D shows that few/no dead cells were present. Additionally, the live cells presented a homogeneous distribution within the 3D-printed structure, confirming the viability of all the designs and validating previous viability and proliferation results.

**Figure 8:**
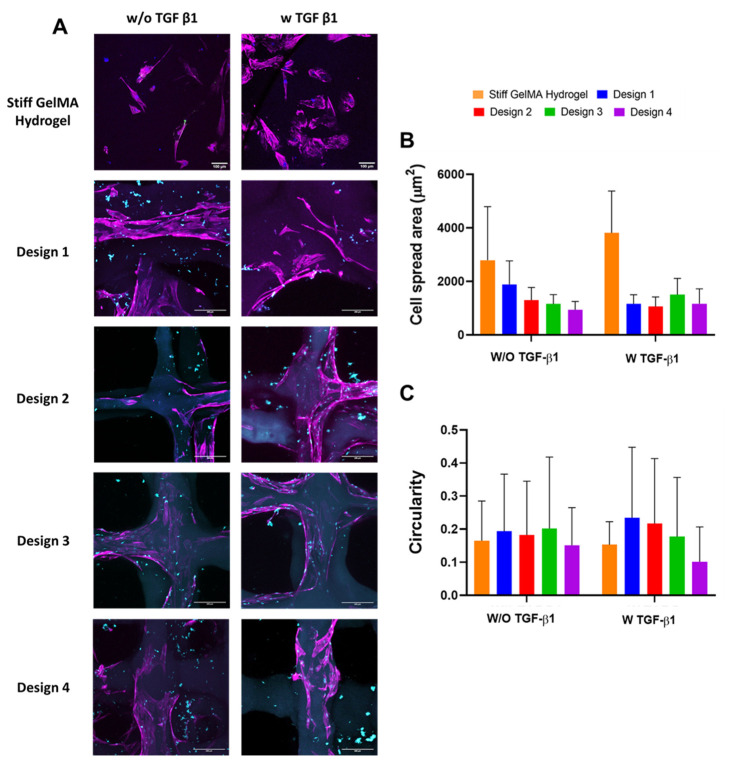
Effect of porous architecture of different 3D-printed scaffold designs on fibroblast infiltration and morphology (**A**), cell spread area (**B**) and circularity (**C**). DAPI (blue) and phalloidin (purple). Scale bar = 100 µm.

In this study, we showed that bulk GelMA hydrogel stiffness (Figure 2) promotes morphological changes in the fibroblasts associated with myofibroblast activation. Recent studies have shown that fibroblasts exhibit quantitative differences in morphology and cytoskeletal architecture following culture in 3D scaffolds versus 2D substrates, with cells presenting a more elongated morphology with increasing pore size [58]. Tolksdorf et al. (2020) demonstrated an anti-fibrotic effect of smaller-size pores in silicone-based substrates [59]. With this in mind, we assessed fibroblast infiltration and morphology within the 3D-printed GelMA scaffold range at 7 days post-seeding, with a stiff GelMA hydrogel with and without TGF-β1 as a positive control. It is important to note that the scaffolds were fabricated using a 10% GelMA concentration, which formed the stiff GelMA hydrogels and promoted the greatest change in fibroblast morphology towards a myofibroblast phenotype. Fibroblasts successfully infiltrated the 3D-printed GelMA scaffolds independent of pore size and TGF-β1 treatment and were observed lining the pores of the scaffolds in all design groups (Figure 8A). We observed an increase in fibroblast elongation within the scaffold pores in all groups compared with the stiff GelMA hydrogel.

**Figure 9:**
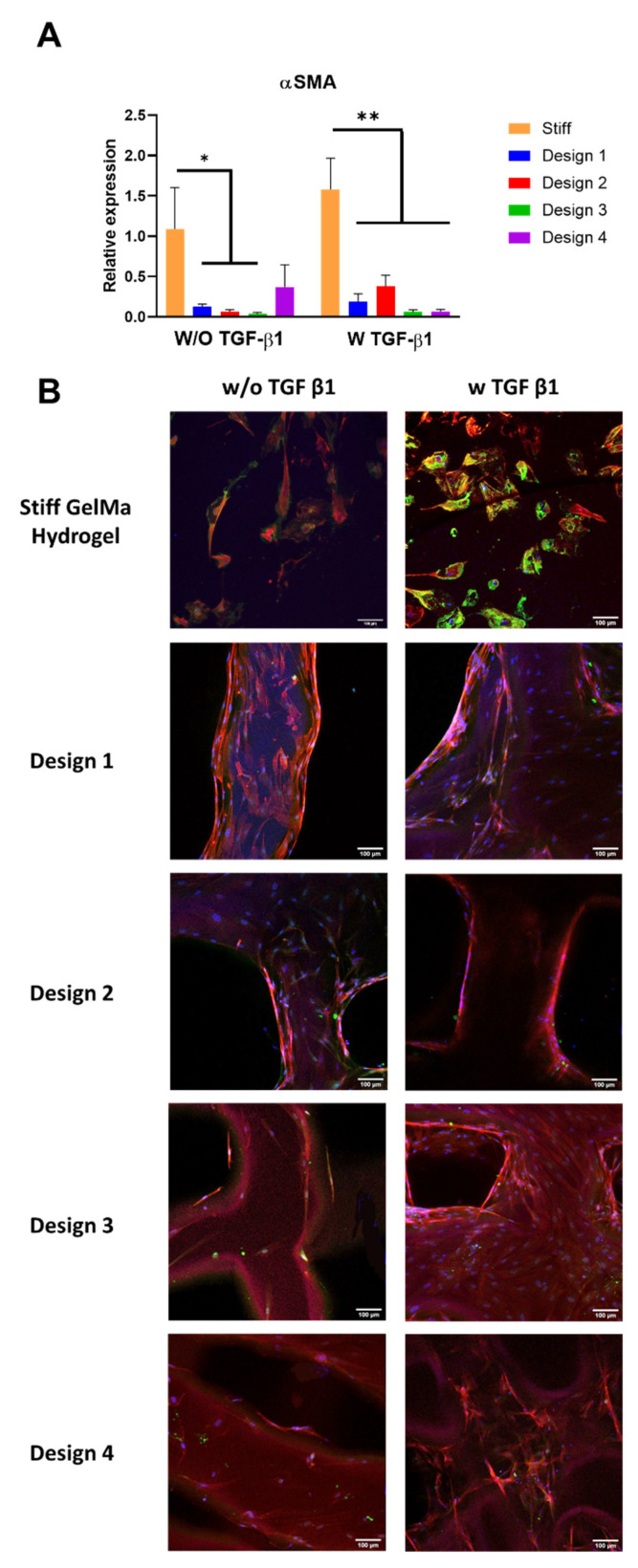
(**A**) Gene expression of αSMA in the 3D-printed scaffold design range compared with the stiff GelMA hydrogel. * *p* < 0.05 compared to the stiff GelMA hydrogel in the absence of TGF-β1; ** *p* < 0.05 compared to the stiff GelMA hydrogel in the presence of TGF-β1. (**B**) αSMA protein expression in the 3D-printed scaffold design range compared to the stiff GelMA hydrogel. DAPI (blue), phalloidin (red) and αSMA (green). Scale bar = 100 µm.

This elongated morphology was similar in all four scaffold designs with and without TGF-β1. Interestingly, the circularity of fibroblasts within the porous scaffolds was similar to that observed on the stiff GelMA hydrogel (Figure 8C). However, the cell spread area was reduced in the porous GelMA scaffolds compared with the stiff GelMA hydrogel, with a significant reduction observed in design 4 (Figure 8B). These results suggest that the change in morphology observed is a direct effect of pore size and the cells’ ability to bridge the pores [58,60] and not indicative of myofibroblast activation. In fact, αSMA gene expression was significantly down-regulated in all four 3D-printed GelMA scaffold designs (*p* < 0.05) compared to the same GelMA concentration in stiff GelMA hydrogels, both with and without TGF-β1 (Figure 9A). These results were confirmed by immunofluorescence, which showed reduced αSMA protein expression in the 3D-printed GelMA scaffolds compared to the stiff GelMA hydrogel, more pronounced in the presence of TGF-β1 (Figure 9B), suggesting that the scaffold’s porous architecture can inhibit the mechano-regulation induction of myofibroblast activation seen in the GelMA hydrogels of similar mechanical properties.

Recently, it has been shown that the presence of pores can reduce both in vitro and in vivo fibrosis by reducing the presence of αSMA and TGF-β1 and therefore by reducing fibroblast activation [61,62]. This effect might be because of the presence of texture on surfaces, the existence of pores, promoting the growth of fibroblasts on the surface, resulting in a decrease in contractile forces and therefore a reduction in fibrotic activity [63,64]. However, to the best of our knowledge, the same effect has not been found on 3D-printed structures.

**Figure 10:**
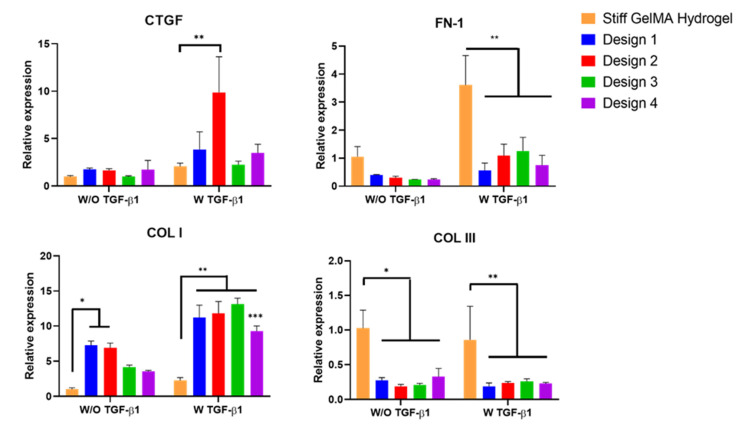
Expression of CTGF, FN, Col I and Col III in four 3D-printed GelMA scaffold designs compared with the bulk stiff GelMA hydrogel at 7 days. * *p* < 0.05 compared to the stiff GelMA hydrogel in the absence of TGF-β1, ** *p* < 0.05 compared to the stiff GelMA hydrogel in the presence of TGF-β1, ** *p* < 0.05 compared to design 3 and *** *p* < 0.05 design 4 in the presence of TGF-β1.

To further elucidate the effect of a GelMA scaffold’s porous architecture on myofibroblast activation, CTGF, FN, Col I and Coll III gene expression were evaluated in the different scaffolds and compared to the stiff GelMA hydrogel (Figure 10). The majority of cells involved in wound healing express TGF-β1, which plays a critical role in ECM production. As such, similar to the GelMA hydrogel stiffness studies, the fibroblast-seeded scaffolds were cultured both with and without TGF-β1. The expression of CTGF in the porous GelMA scaffolds was comparable to the stiff GelMA hydrogel in the absence of TGF-β1, with no statistical difference found between the four scaffold designs. Interestingly, when TGF-β1 was added to the culture media, a significant increase in CTGF expression (*p* < 0.05) was observed in design 2 scaffolds compared with the stiff GelMA hydrogel and other scaffold groups. Studies on other cell types have revealed the important roles of CTGF in the TGF-β1-dependent induction of ECM production and myofibroblast differentiation [65,66,67]. 

The levels of main structural components of skin ECM, such as Col I and III and FN, are all increased in raised dermal scar tissue [68]. We observed increased expression of these ECM molecules in the medium and stiff GelMA hydrogels compared with the soft GelMA hydrogel as a result of increasing mechanical stiffness. However, the porous architecture of the 3D-printed GelMA porous scaffolds significantly altered the expression profiles in comparison to the non-porous stiff GelMA hydrogels. Fibroblasts found in dermal scar tissue have a rate of FN-1 biosynthesis that is four times as high as that of fibroblasts in the normal dermis [69]. Yet, Figure 10 shows that FN gene expression was down-regulated in all four porous scaffold groups, independent of pore size, compared with the non-porous stiff GelMA hydrogel. The addition of TGF-β1 significantly up-regulated FN-1 expression in the stiff GelMA hydrogels and resulted in a more pronounced pore size effect on FN-1 expression within the GelMA scaffold range, with an increase in expression observed in designs 2 and 3. Nonetheless, even in the presence of TGF-β1, FN-1 expression was significantly down-regulated in all four scaffold designs (*p* < 0.05) compared with the stiff GelMA hydrogel. During the early stages of wound healing, myofibroblast expression of Col III is greater than that of Col I [1]. Our results showed that, similarly to FN-1 expression, Col III gene expression was significantly down-regulated in all four porous GelMA scaffold designs with different pore sizes and porosities (*p* < 0.05) compared with the stiff GelMA hydrogel. This result, although unexpected, could be related to the way cells sense the stiffness in the non-porous and porous structures. 

This down-regulation was observed in both treated and untreated TGF-β1 groups. Conversely, Col I gene expression was up-regulated in design 1 and 2 scaffolds (*p* < 0.05) when compared with the stiff GelMA hydrogel. However, with the addition of TGF-β1, Col I was significantly up-regulated in all four scaffold designs compared with the stiff GelMA hydrogel, although the up-regulation of Col I in design 4 was significantly less than that observed in designs 1, 2 and 3. Taken together, these results show that the porous architecture of 3D-printed GelMA scaffolds promotes gene expression of Col I, a key structural protein in skin, while inhibiting TGF-β1-induced overexpression of FN and Col III. In contrast, collagen I gene expression was up-regulated. Nevertheless, Webb et al. cultured fibroblasts on porous surfaces, and after 4 weeks, they observed collagen type I and fibronectin filling the pores [70]. Taking all this into consideration, the increase in collagen type I gene expression in our study might suggest that cells produce collagen type I to fill in the gaps in our 3D-printed scaffolds.

Nevertheless, further studies are required to fully understand the specific interplay between a GelMA scaffold porous architecture and mechanical properties in terms of fibroblast-to-myofibroblast transition and determine whether the scaffolds developed can improve dermal skin wound healing in vivo. 

## 4. Conclusions

In this study, we demonstrated for the first time that the mechanical properties of GelMA hydrogels have a significant effect on human dermal fibroblast behaviour, whereby increasing stiffness promotes myofibroblast activation through increased fibrosis-related gene and protein expression. While not optimal for myofibroblast inhibition, we used the same concentration of GelMA (10%) as the stiffest non-porous hydrogel and successfully developed a range of 3D-printed GelMA scaffolds with a tuneable porous architecture and porosity that facilitated fibroblast viability, proliferation and infiltration. We further demonstrated that the introduction of pores into the GelMA stiff hydrogel negates the effect of bulk GelMA hydrogel stiffness on myofibroblast activation, with reduced expression of αSMA, FN and Col III observed within scaffold groups treated with TFG-β1. Moreover, Col I significantly increased in scaffolds treated with TGF-β1. Collectively, these results show that these 3D-printed GelMA scaffolds have the potential to improve dermal skin regeneration, whilst inhibiting fibrosis and scar formation.

## Figures and Tables

**Table 1:** List of gene transcripts analysed by qRT-PCR; Qiagen Quantitect-validated primers were used to analyse gene expression levels of target proteins.

Target Proteins	Target Gene Reference	Catalogue Ref.
Collagen I (COL1A1)	Hs_COL1A1_1_SG	QT00037793
Collagen III (COL3A1)	Hs_COL3A1_1_SG	QT00095431
Fibronectin I (FN1)	Hs_FN1_1_SG	QT00038024
Connective tissue growth factor (CTGF)	Hs_CTGF_1_SG	QT00052899
Alpha smooth muscle actin (αSMA)	Hs_ACTA2_1_SG	QT00088102
GAPDH	Hs_GAPDH_1_SG	QT00079247

**Table 2:** 3D printing parameters used to produce all designs.

	Needle Gauge	Number of Layers	Layer Height (µm)	Perimeter (mm)	Printing Velocity (mm·mm^−1^)	Cross-linking Time (min)	Cross-Linking Intensity (MV/cm^2^)
**All designs**	30	12	150	10	340	3	10

## Data Availability

Not applicable.
